# Endothelial to mesenchymal cell transition in diabetic retinopathy: targets and therapeutics

**DOI:** 10.3389/fopht.2023.1230581

**Published:** 2023-09-07

**Authors:** Wasef Nijim, Mohamed Moustafa, Julia Humble, Mohamed Al-Shabrawey

**Affiliations:** ^1^ Medical College of Georgia, Augusta University, Augusta, GA, United States; ^2^ Eye Research Center, Oakland University William Beaumont School of Medicine, Rochester, MI, United States; ^3^ Eye Research Institute, Oakland University, Rochester, MI, United States; ^4^ Foundation Medical Studies, Oakland University William Beaumont School of Medicine, Rochester, MI, United States

**Keywords:** diabetic retinopathy, endothelial-mesenchymal transition, PDR, diabetic macular edema, retinal detachment, therapeutic targets, NPDR

## Abstract

Diabetic retinopathy (DR) is a result of neurovacular insults from hyperglycemia in diabetes mellitus (DM), and it is one of the top causes of vision loss throughout the modern world. This review article explores the role endothelial to mesenchymal transition (EndMT) has on the pathogenesis of DR. EndMT contributes to the disruption of the blood-retinal barrier, vascular leakage, neovascularization, and fibrosis observed in DR. Risk factors and biomarkers associated with DR severity are discussed, highlighting the importance of early detection and targeted therapies. Current treatments primarily focus on anti-vascular endothelial growth factor (anti-VEGF) agents, corticosteroids, and laser photocoagulation. However, emerging therapeutic strategies aimed at inhibiting EndMT and its downstream effects show promise in preventing the development and progression of DR. Understanding the molecular and cellular mechanisms underlying EndMT in DR provides valuable insights into the disease process and offers potential options for the development of potential treatments.

## 1 Epidemiology

Diabetic Retinopathy (DR) is a microvascular complication of diabetes mellitus (DM), caused by hyperglycemic insults leading to vision-threatening damage to the retina (1). DR is the number one cause of loss of vision in adults within the United States (2). Early detection and immediate treatment are paramount in avoiding blindness from this common disease (2).

In the next ten years, the global prevalence of DM has been estimated to become 592 million (3). DR is the most common microvascular complication of diabetes (4). Ninety-three million people worldwide suffer from DR, with 25-30% of these people having vision-threatening complications (5). Of these patients, 5-8% of patients require laser treatment, and 5% require vitrectomy surgery (6).

Hyperglycemia and subsequent metabolic disorders activate inflammatory and oxidative pathways within the retina that cause damage to retinal vessels. Growth of microaneurysms and breakdown of the blood-retinal barrier (BRB) and subsequent development of diabetic macular edema (DME) and hard exudates are the typical clinical manifestations of non-proliferative diabetic retinopathy (NPDR) (1). Vasoconstriction and vascular occlusion within retinal capillaries lead to the development of ischemic pale spots ‘cotton-wool spots’ (2). Development of severe retinal ischemia eventually causes the upregulation of angiogenic factors, like Vascular Endothelial Growth Factor (VEGF) (3). The upregulation of angiogenic factors causes the characteristic neovascularization seen in proliferative diabetic retinopathy (PDR) (3).

### 1.1 Risk factors and biomarkers

DR affects patients with diagnosed or undiagnosed DM. The most significant risk factors for developing DR are older age, longer duration of DM, high glycemic levels, and hypertension (2). A higher HbA1C is significantly associated with advancement of DR, whereas intense glycemic control significantly correlates with a reduction in incidence and progression of DR (7, 8). Recent studies have shown high variability in glucose levels also contributes to severity of DR; therefore, controlling post-prandial glucose levels is important in reducing the progression of DR as well (9, 10). Tight blood pressure control is another important risk factor modification that has been shown to reduce the damage inflicted on the retina from DR (11). Other modifiable risk factors that are not as critical in DR but still contribute to the development and advancement of disease include: nephropathy, hyperlipidemia, smoking, and obesity (12, 13). Non-modifiable risk factors include pregnancy, age, and genetics (1, 14).

Despite the above risk factors, current studies find a variation between the development and severity of DR that cannot be fully explained by the beforementioned risk factors. Therefore, identifying newer biomarkers that can help stratify patients and determine their response to therapeutics is important (2). Systemic biomarkers that have been found to strongly correlate with severity of DR include markers for inflammation, such as C-reactive protein (CRP), homocysteine, and advanced glycation end products (AGEs) (15–17). Newer markers discovered include apolipoprotein, vitamin D, leptin, as well as various genetic markers (1). Ocular biomarkers from sampling vitreous and tears include VEGF and platelet-derived growth factor (PDGF), but these markers need more validation studies for predicting DR severity (18, 19).

### 1.2 Mechanisms of vision loss

DME is the most common cause of visual loss in DR patients occurring in both NPDR and PDR at various levels of disease (3, 20). DME results from leakage of fluid and proteins from retinal vessels into the macula due to breakdown of BRB (20). Hard exudates and edema can be seen on fundoscopy (21). Tractional retinal detachment (TRD) with vitreous hemorrhage is the second form of vision loss resulting from DR (20). Unlike DME, TRD only occurs in PDR due to the fibrovascular scarring generated during neovascularization being pulled on by the vitreous (3). For TRD, a ring of white scar tissue can be seen on fundoscopy (22). Fibrovascular proliferation can also cause vitreous hemorrhage, contributing to vision loss from PDR (20).

Endothelial cell to Mesenchymal Transition (EndMT) occurs during the various stages of NPDR and PDR (23). The transformation can contribute to both mechanisms of vision loss mentioned above. EndMT contributes to DME by disrupting the BRB, increasing vascular permeability due to the loss of endothelial characteristics (24). This disruption leads not only to leakage of fluid into the retina, but also of inflammatory mediators (24). EndMT contributes to fibrosis within the retina due to the gain of mesenchymal characteristics and eventually increases the likelihood of TRD during PDR (23).

### 1.3 Pathogenesis of microvascular dysfunction in diabetic retinopathy

There are several metabolic pathways involved in the pathogenesis of DR, such as the polyol, protein kinase c (PKC), and hexosamine pathways (25). Activation of these pathways cause upregulation of cytokines and growth factors that leads to increased vascular permeability and occlusion of blood vessels (1). Initially, the blood vessels within the retina dilate in response to the hyperglycemic insults to increase retinal metabolism (20).

Pericytes are specialized, contractile mesenchymal stem cells (MSCs) that provide vascular tone and perfusion pressure within the capillaries of the retina (26). Hyperglycemia causes apoptosis of pericyte and endothelial cells leads to microaneurysm formation, breakdown of BRB, and development of retinal ischemia (20). Insufficient circulation leads to upregulation of VEGF (27), the primary angiogenic factor that is associated with the vascular permeability and angiogenesis seen in DR (20).

The high metabolic rate of the neural retina requires the regulation of a distinct supply of specific nutrients by the BRB (28). It regulates the transport of ions, water, and nutrients and prevents immune cells and antibodies from passing into the retina tissue (29). The BRB includes the inner BRB (iBRB) and the outer BRB (oBRB) (30). The iBRB consists of endothelium, Müller cells, and pericytes (30). The capillaries of the iBRB provide oxygen, glucose, and other nutrients to neurons and prevent other molecules from entering the retina for protection (31). The oBRB is composed of retinal pigment epithelium (RPE) and functions to regulate the transport between the choriocapillaris and the retina (32). Various growth factors and cytokines that are upregulated in DR, such as VEGF, HIF-1, and IL-1 β, induce BRB breakdown through various mechanisms (33). Damage to the iBRB and oBRB leads to fluid and protein extravasation into the macula, as well as the degeneration of retinal capillaries, playing a critical role in the development of DME and severe visual loss in DR (32).

**Figure 1:**
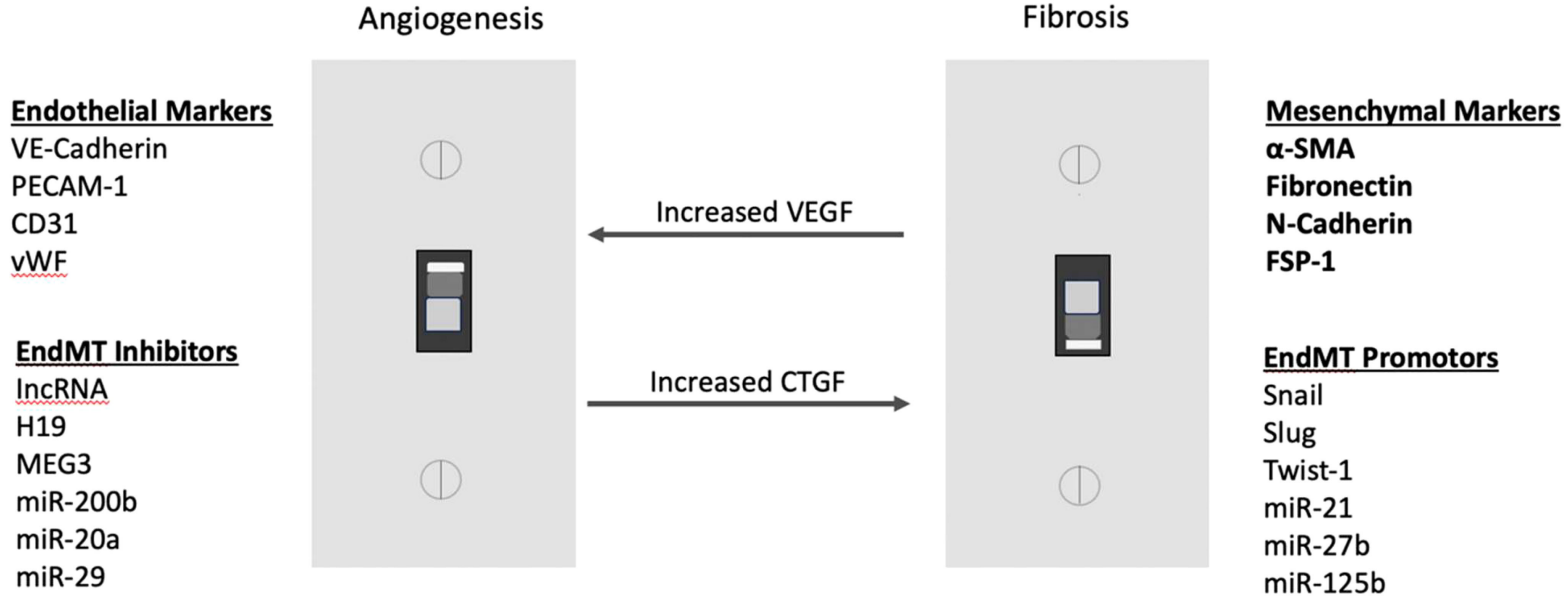
Angiofibrotic switch: elevated CTGF over vascular VEGF promotes fibrosis in PDR.

One of the advanced stages of DR, known as PDR, is characterized by neovascularization, as well as an increase in the presence of myofibroblasts (34). This process can lead to the development of fibrovascular tissue, which can result in traction on the retina, leading to TRD and eventual blindness (2). There is a switch at some point in PDR between neovascularization and fibrosis; this is known as the angio-fibrotic switch (34). As previously stated, VEGF is majorly responsible for neovascularization (20). Connective tissue growth factor (CTGF) is another factor upregulated during DR that contributes mainly to fibrosis within the eyes of patients (34). The decrease in VEGF and increase in CTGF is associated with the switch from angiogenesis to fibrosis in PDR (**Figure 1**) (34). CTGF is a Transforming Growth Factor-Beta (TGF-β) effector that induces tissue fibrosis during PDR (35). In addition to fibrosis during PDR, TGF-β is also a main contributor to EndMT earlier on in DR (23).

As previously stated, the early vascular loss in DR is a major contributor to the clinical manifestations of DR, and endothelial cell and pericyte apoptosis is only partly responsible for this vascular loss (20). Another part of the vascular loss found in DR is the transformation of endothelial cells to mesenchymal cells through EndMT (23). As a consequence of EndMT, endothelial cells undergo a shift in their cell markers and phenotype, transitioning from their original characteristics to acquire the cell markers and phenotype typically associated with mesenchymal cells (36–38). This process leads to the breakdown of the BRB and contributes to the microvascular pathogenesis of DR (23).

### 1.4 Current treatments

In addition to optimizing blood glucose, lipids, and blood pressure in diabetic patients, there are various intraocular management strategies that have become standard practice in DR patients (39, 40). For instance, the management of DME has been significantly altered with the introduction of intravitreal anti-VEGF injection therapies, such as ranibizumab, bevacizumab, and aflibercept (2). Several randomized control trials since 2010 have shown these agents significantly reduce DME and improve vision (41–45). Aflibercept has been found to be the most efficacious agent in patients with initial poor visual acuity (46). Despite all the current research on anti-VEGF treatments, the optimal frequency of injections and total time for treatment courses is still unknown (2). The current practice is to administer multiple injections within the first year of treatment and then a gradual decrease in the following years to maintain remission (39). Intravitreal anti-VEGF injections have also been shown to benefit patients with PDR (47). These medicines do, however, have some limitations and adverse effects (20). The short half-life of these agents means that initially, bimonthly injections are necessary to ensure efficacy (20). Elevation of intraocular pressure, vitreous hemorrhage, and inflammation are uncommon adverse effects (20). A rare adverse effect due to the high number of injections necessary is endophthalmitis (20, 48). Additionally, high cost and poor patient adherence are of concern when it comes to this treatment modality (20). Currently, there is research being done on other various anti-angiogenic agents that inhibit vasoconstriction and vascular occlusion within retinal capillaries multiple angiogenic factors in addition to or other than VEGF, such as Squalamine and Nesvacumab, respectively (20, 49).

In cases of refractory DME that are unresponsive to anti-VEGF treatments, intravitreal corticosteroid injections can be used (50). These refractory cases are thought to be driven by the effects of multiple cytokines (20). Corticosteroids target inflammatory mediators known to contribute to the pathogenesis of DME (20). There have been multiple clinical trials done for DME treatment using triamcinolone acetonide, dexamethasone (DEX) intravitreal implant, and fluocinolone intravitreal implant (48, 51–53). The use of intravitreal corticosteroids has been associated with a lower number of injections, lower cost, and higher compliance (20). However, given the higher incidence of adverse effects, such as cataracts, glaucoma, and vitreous hemorrhage, and no proven benefit in PDR, intravitreal corticosteroid injections are considered second-line to anti-VEGF injections (20, 48, 51–53).

On the other hand, in patients that have progressed to PDR, panretinal laser photocoagulation (PRP) is considered first-line due to its effectiveness in reducing vision loss in this patient population, especially in patients with vitreous hemorrhage complications (54–56). Aflibercept, an anti-VEGF agent previously mentioned, has been shown to result in better visual outcomes and is a safer alternative to PRP in select patients (47). Due to laser-induced retinal damage from PRP, adverse effects include mild central visual acuity loss and reduced night vision (57). However, PRP remains important as an adjuvant and rescue therapy for PDR patients with high-risk complications by reducing the rate of severe visual loss and halting the progression of retinopathy (20). In modern times, there have been efforts to develop newer laser approaches that reduce side effects (20) e.g. the pattern scanning laser (PASCAL) (58), the subthreshold micropulse diode laser (D-MPL) (59), and the navigated laser system (NAVILAS) (60).

Other treatments for DR under clinical evaluation include cardiolipin inhibitors, mitochondria-specific antioxidants, as well as other classes of antioxidants (61–63). All these treatments for DR are focused on preventing late complications of the disease. An alternate approach being investigated currently is treating the root causes and early developments within DR (23). We researched the current literature on EndMT, an early pathological event in DR and potential treatments to prevent it and subsequent complications.

## 2 EndMT

EndMT is a process where endothelial cells undergo a phenotypic transition to mesenchymal cells (64). This process has been implicated in the pathogenesis of various diseases, including diabetic complications (23). EndMT in diabetes is prompted by various factors, such as elevated blood sugar levels and AGEs (23).

At the cellular level, EndMT is identified by the disappearance of endothelial markers such as CD31, while gaining mesenchymal markers such as α-smooth muscle actin (α-SMA) (65). This process is driven by various pathways, including TGF-β, Notch, and Wnt/β-catenin signaling pathways (65, 66). Furthermore, hyperglycemia can promote EndMT by triggering NF-κB signaling (67, 68). These signaling pathways ultimately lead to the increased expression of transcription factors, such as Snail, Slug, and Twist that promote EndMT (69–72). Additionally, epigenetic regulation, such as DNA methylation and histone modification, plays a role in EndMT (23, 73). One post-translational complex, methyltransferase-like 3 (METTL3), has been recently found to play an important role in EndMT. METTL3 is responsible for the N6-methyladenosine (m6a) modification, where a methyl group is added to the sixth nitrogen of adenosine in RNA. METTL3 adds the m6a modification to transient receptor potential cation channel 6 (TRPC6), which works through the calcineurin/NFAT pathway, to increase mesenchymal marker α-SMA and decrease endothelial markers CD31 and VE-cadherin (74). Another epigenetic process involved in EndMT is the methylation of maternally expressed gene 3 (MEG3) by DNA methyltransferase 1 (DMT1). Recent research has shown that MEG3 is downregulated in rat models of DR due to methylation by DMT1. Inhibition of MEG3 activates the PI3K/AKT/mTOR pathway, which is implicated in the pathogenesis of DR and EndMT (75). MicroRNAs (miRNAs) have also been shown to regulate the expression of EndMT-related genes (24). In diabetes, the expression of certain miRNAs is altered, which can promote EndMT (24, 76).

The impact of EndMT on vascular function in diabetes is multifactorial. One of the key consequences of EndMT is the loss of function of the BRB, leading to increased vascular permeability and the leakage of unwanted proteins and immune cells into the retina, causing edema and inflammation of the surrounding tissues (77, 78). EndMT also contributes to the buildup of extracellular matrix (ECM) proteins like collagen, forming fibrotic tissue (24). In DR, this excess of ECM proteins leads to the enlargement of the basement membrane in the retina. This process is part of the pathogenesis of increased vascular permeability in DR (79).

### 2.1 EndMT in diabetic retinopathy

DR, a leading cause of visual impairment in adults, can be categorized into NPDR and PDR stages (20). EndMT occurs throughout these stages, contributing to increased vascular permeability and fibrovascular scarring (23). Endothelial cells within retinal capillaries help maintain the BRB, which consists of cells joined together to prevent certain substances from entering the retina from the circulatory system (80). EndMT disrupts the integrity of the BRB, permitting plasma and proteins to enter the retina, which can give rise to complications like DME (80, 81).

In DR, EndMT leads to thickening of the basement membrane and increased deposition of matrix proteins, contributing to the breakdown of the BRB (24, 82). Additionally, EndMT increases the number of myofibroblasts, which can lead to TRD because of an increase in fibrosis within the retina (79). Hyperglycemia, suppression of long non-coding RNAs (lncRNAs), upregulation of Notch2, and various circRNAs and miRNAs are all implicated in EndMT induction through TGF-β signaling in DR (76, 81). Overexpression of H19 and MEG3, effectively prevents EndMT (24, 75, 81). Inhibition of Notch2 and suppression of various circRNAs are also potential therapeutic targets for preventing EndMT in DR (76).

**Figure 2:**
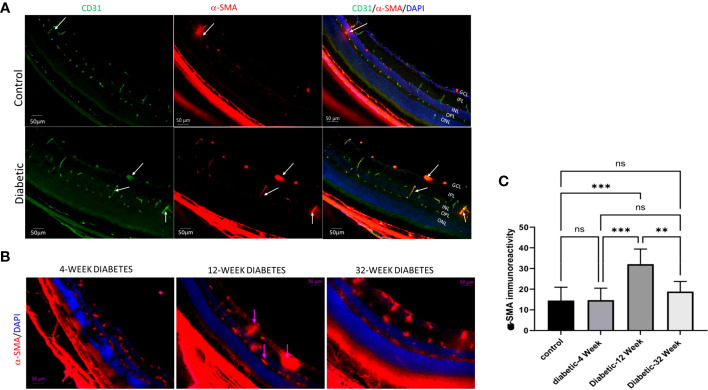
**(A)** Immunofluorescence of α-SMA in retinal cryosections of 12-Week diabetic vs. Control (10X magnification). **(B)** 4 Week Diabetic vs. 12 Week Diabetic vs. 32 Week Diabetic α-SMA Immunoreactivity in retinas of diabetic mice (20x magnification). **(C)** One-way ANOVA multiple comparison test of α-SMA immunoreactivity intensity for 12 Week Control Group, 4 Week Diabetic, 12 Week Diabetic, and 32 Week diabetic mice. (Scale bar= 50μm). **P<.005, ***P<.0005, and ns, not significant.

We conducted our own study on EndMT within DR. The goal of our study was to evaluate if there is a significant difference in the amount of EndMT at different stages of the disease. EndMT and fibroproliferative transformation were characterized by *de novo* cellular expression of α-SMA. We used Ins2Akita mice as an experimental model of type 1 diabetes. The mice eyes were dissected to retrieve their retinas. We then performed immunofluorescence (IF) on the retinas of 23 diabetic mice at 4-weeks (8 mice), 12-weeks (8 mice), and 32-weeks (7 mice) old. We also performed IF on the retinas of 9 non-diabetic mice that were 12-weeks-old to use as a control. The antibodies used to stain the retina slides were for α-SMA, CD31, and DAPI. These were used as markers for mesenchymal tissue, endothelial cells, and nuclear markers, respectively. Images of these slides were taken at 20x using fluorescence microscopy. The intensity of α-SMA was analyzed using Image J software, and statistical analysis was performed using One-way ANOVA Multiple Comparisons Test with a p-value <0.05 considered significant. An increase in α-SMA intensity correlated with an increase in EndMT. Our results indicated that there was a significant increase in EndMT in the retina of diabetic mice compared to the control group (**Figures 2A, C**). Moreover, EndMT was significantly increased in the 12-week-old diabetic mice when compared to the 4-week-old and 32-week-old diabetic mice (**Figures 2B**, **C**). . There was no significant difference between the amount of EndMT when comparing the 4-week-old diabetic mice with the 32-week-old diabetic mice (**Figure 2C**). The significant increase of EndMT, at 12-weeks in comparison to 4-weeks may be due to a temporal requirement for diabetes to initiate the process of EndMT. The significant decline of EndMT, at 32-weeks in comparison to 12-weeks, could be attributed to the loss of pericytes and endothelial cells by 24 weeks in DR (83). Pericytes are mesenchymal stem cells, so they contribute to the increase in mesenchymal cell population found with EndMT (83).

**Figure 3:**
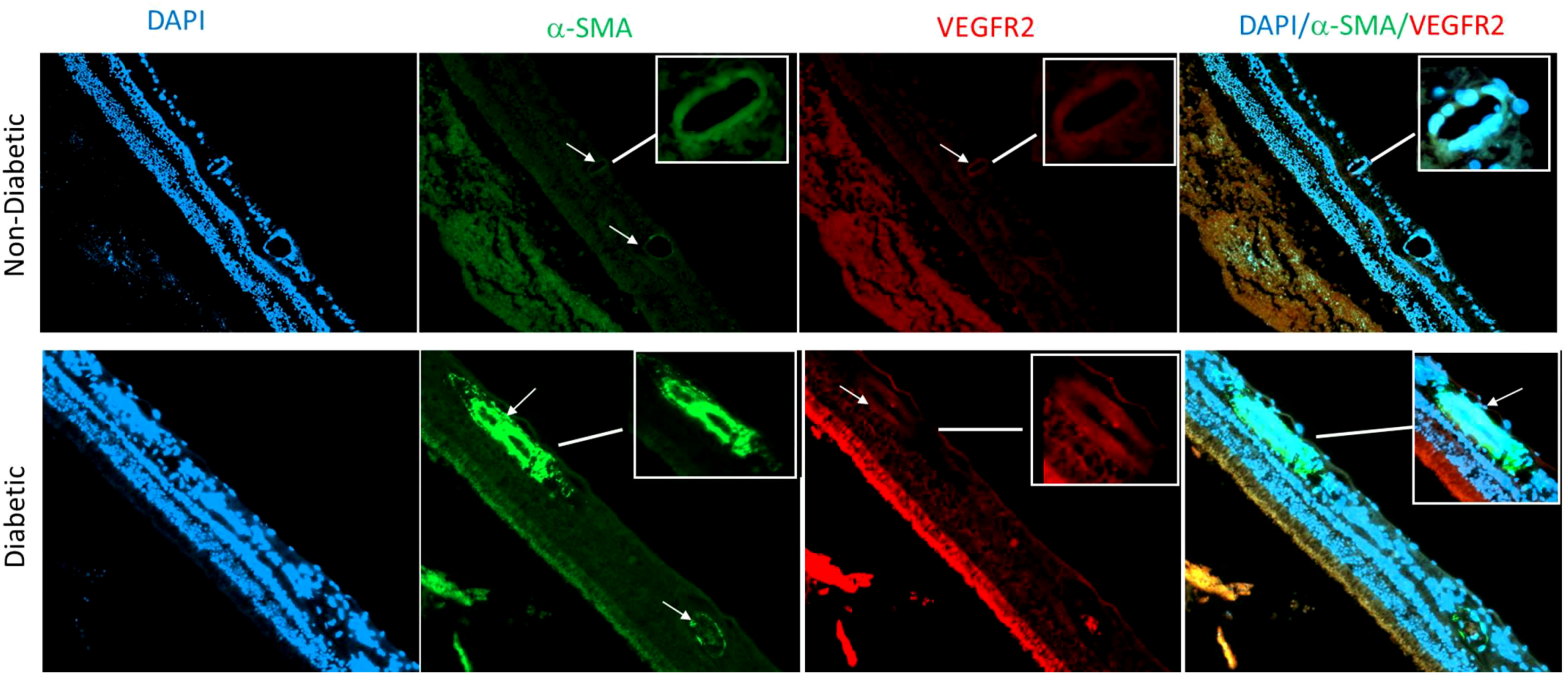
Immunofluorescence of α-SMA in retina sections of human subjects using anti- α SMA (green), vascular marker VEGFR2 (red) and nuclear marker DAPI (blue). There is an obvious increase in α-SMA immunoreactivity in retinal vessels (arrows) of diabetic human donors compared to the non-diabetic donors.

Our findings are consistent with the suggestion that EndMT plays a crucial role in the pathogenesis of microvascular dysfunction in DR. This transformation could lead to loss of endothelial cells, capillary degeneration, vascular leakage, and retinal hyperpermeability, leading to complications like DME and TRD. Our study provides valuable insight into the temporal changes of EndMT in the context of diabetic retinopathy and highlights the importance of further research in this area. Our research group has started investigating EndMT in postmortem human retina that were obtained from Georgia Eye Bank and processed to prepare paraffin-embedded sections. Slides from 3 diabetic human samples and 3 non-diabetic human samples were used in an IF experiment, with α-SMA as a mesenchymal marker, DAPI as a marker for the nucleus, and VEGFR2 marking endothelial cells. Our preliminary findings suggest that diabetic human retinas exhibit a higher amount of mesenchymal tissue compared to control groups as shown by a marked increase in α-SMA immunoreactivity in retinal vessels of diabetic human subjects compared to the non-diabetic group (**Figure 3**). Further research is needed to confirm these results and to explore the temporal changes of EndMT in diabetic human retinas.

## 3 Targets to prevent EndMT

Exploring therapeutic targets for preventing EndMT in diabetic patients is important, and it involves researching various molecules and pathways. While the exact interplay and regulation between these pathways are not yet fully understood, several potential targets have emerged based on extensive research.

Among the pathways implicated in hyperglycemia-induced EndMT, inhibition of Notch, canonical TGF-β, or non-canonical TGF-β signaling alone has shown promise in preventing EndMT in the context of DR (23). However, understanding the extent of pathway overlap and co-regulation remains a subject of ongoing investigation. Intriguingly, a few medications usually used for glucose control in diabetics, such as sodium-glucose co-transporter 2 (SGLT2) inhibitors and GLP-1 agonists, have also shown effects in suppressing EndMT in different organs (84–86). These medications activate AMP-activated protein kinase (AMPK), which inhibits intracellular TGF-β signaling (85–87).

Sprouty-related proteins with EVH1 domain (SPRED2), a member of the Sprouty/SPRED family, areis known for theirits negative regulation of the Ras/Raf/ERK/MAPK signaling pathway. SPRED 2 is another emerging target under investigation as demonstrated by a study exploring its role on EndMT in DR. The researchers used diabetic rat models and human retinal endothelial cells (HRECs) treated with high glucose to simulate DR. The results showed that SPRED2 expression was reduced in the retinal tissues of diabetic rats and high glucose-treated HRECs. By increasing the levels of SPRED2, the researchers observed a suppression of endothelial injury, inhibition of EndMT by regulating specific markers, improvement of tight junction components, and downregulation of the MAPK signaling pathway. These findings suggest that SPRED2 could be a promising therapeutic target for managing the progression of DR (88).

Other proteins, such as Raf kinase inhibitor protein (RKIP), also play a role in EndMT within DR. Increased RKIP levels have been found to exhibit inhibitory effects on the cellular processes associated with EndMT. Moreover, RKIP downregulation has been found to reduce expression of endothelial markers CD31 and vWF in HRCECs under glucose-induced conditions, while RKIP overexpression resulted in their upregulation. These results suggest that RKIP exerts its action by negatively regulating glucose-induced EndMT and associated cellular events in HRCECs, thereby indicating finding ways to increase RKIP can be a potential therapy for managing EndMT in DR (89).

Additionally, the involvement of LPA-1, a receptor for lysophosphatidic acid (LPA), is being studied as a therapeutic target for EndMT in DR. One study demonstrated that the downregulation of acylglycerol kinase (AGK), an enzyme involved in LPA production, suppressed EndMT in HRECs by modulating the LPA-1/TGF-β/Notch signaling pathway. These results suggest that targeting LPA-1 and its associated signaling pathways may hold therapeutic potential for managing EndMT in the context of DR (90).

Furthermore, recent research has established the role of formyl peptide receptor 2 (FRPR2) in DR, contributing to both pathological neovascularization and EndMT. FRPR2 is a G-protein coupled receptor expressed in a variety of cells, including endothelial cells and glial cells. An *in vitro* study showed that high glucose upregulates FRPR2 in human endothelial cells. They also found that in FRPR2 knock-out diabetic mice, there was a significant decrease in mesenchymal markers on retinal endothelial cells compared to wild-type diabetic mice. This shows that FRPR2 plays an important role in EndMT and may have potential as a novel therapeutic target for DR (91).

Select dietary supplements have displayed potential in inhibiting EndMT. Notably, supplements like resveratrol and eicosapentaenoic acid have shown inhibitory effects on the PKC pathway, effectively impeding the induction of TGF-β and endothelin-1 (ET-1), and subsequently suppressing EndMT in retinal and glomerular endothelial cells, respectively (92, 93). In addition to small molecule compounds, nucleic acid-based approaches have also been explored. Short interfering RNAs (siRNAs) targeting pro-EndMT genes and lncRNAs that mimic anti-EndMT microRNAs have shown promise in preventing EndMT. Experimental silencing of lncRNAs ZFAS1 and MALAT1, as well as induction of various miRNAs, have proven to be effective in suppressing hyperglycemia-induced EndMT through different molecular pathways (76, 94–98). Moreover, synthetic lncRNAs have emerged as a potential strategy to suppress glucose-induced EndMT (99).

Experimental upregulation of lncRNAs H19 and MEG3, which are inhibited by glucose, has shown promise in preventing EndMT in specifically DR (81, 100). Moreover, emerging evidence suggests that specific members of the bone morphogenetic protein (BMP) family (BMP2, BMP4, and BMP9), a subgroup of the TGF-β superfamily, play crucial roles in the regulation of the EndMT process in DR (101, 102). Targeting the inhibitory modulation of these BMPs represents a promising therapeutic approach for preventing or attenuating EndMT in DR.

**Figure 4:**
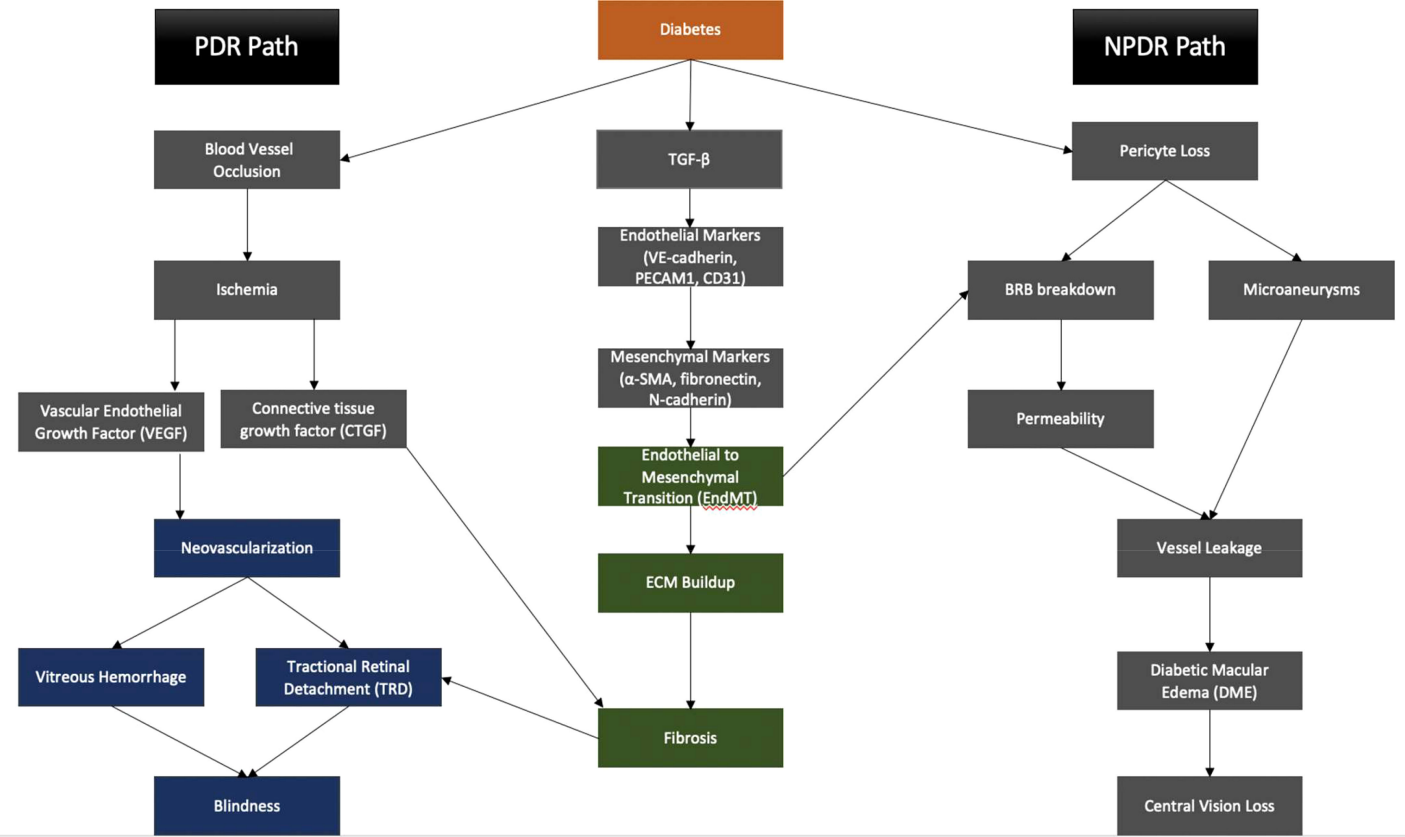
EndMT contributes to the pathogenesis of PDR and NPDR, eventually leading to central vision loss and blindness.

**Figure 5:**
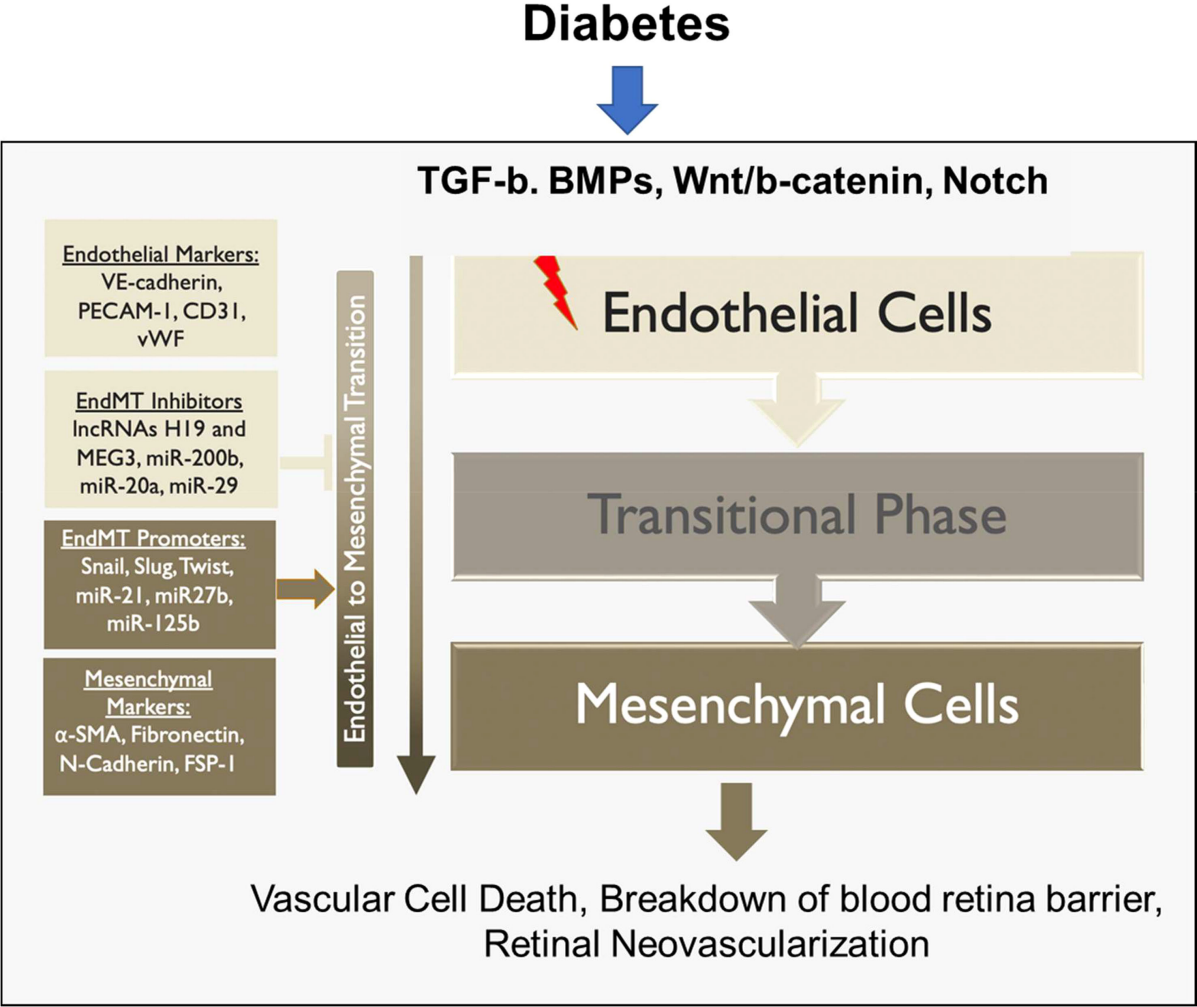
Role of EndMT in the pathogenesis of diabetes-induced retinal microvascular dysfunction.

In summary, EndMT exerts its influence on both the non-proliferative and proliferative stages of DR, contributing to the mechanisms underlying blindness in DR (**Figure 4**). The critical role played by EndMT in the development of microvascular dysfunction in DR (**Figure 5**) suggests the need for further investigations to understand the underlying molecular and cellular mechanisms. This should lead to the identification of new therapeutic strategies to mitigate the early pathological changes in DR and preserve vision of diabetic patients.

## Author contributions

WN – Conducted lab experiments mentioned in manuscript, wrote full initial draft and created figures of manuscript, revised and edited manuscript. JH – Revised and edited manuscript, conducted lab experiments mentioned in manuscript. MM – Conducted lab experiments mentioned in manuscript. MA-S – Conception of lab experiments mentioned in manuscript, conducted lab experiments, revised, and edited manuscript. All authors contributed to the article and approved thesubmitted version.
